# Focused ultrasound thalamotomy for tremor treatment impacts the cerebello-thalamo-cortical network

**DOI:** 10.1038/s41531-023-00543-8

**Published:** 2023-06-15

**Authors:** Louisa Dahmani, Yan Bai, Meiling Li, Jianxun Ren, Lunhao Shen, Jianjun Ma, Haiyang Li, Wei Wei, Pengyu Li, Danhong Wang, Lei Du, Weigang Cui, Hesheng Liu, Meiyun Wang

**Affiliations:** 1 Department of Medical Imaging, Henan Provincial People’s Hospital & People Hospital of Zhengzhou University, Zhengzhou, China; 2 Athinoula A. Martinos Center for Biomedical Imaging, Department of Radiology, Massachusetts General Hospital, Harvard Medical School, Charlestown, MA 02129 USA; 3 Department of Neurology, Henan Provincial People’s Hospital & People Hospital of Zhengzhou University, Zhengzhou, China; 4 Department of Neurosurgery, Henan Provincial People’s Hospital & People Hospital of Zhengzhou University, Zhengzhou, China; 5 Changping Laboratory, Beijing, China; 6 Biomedical Pioneering Innovation Center, Peking University, Beijing, China

**Keywords:** Parkinson's disease, Parkinson's disease

## Abstract

High-intensity Magnetic Resonance-guided Focused Ultrasound (MRgFUS) is a recent, non-invasive line of treatment for medication-resistant tremor. We used MRgFUS to produce small lesions in the thalamic ventral intermediate nucleus (VIM), an important node in the cerebello-thalamo-cortical tremor network, in 13 patients with tremor-dominant Parkinson’s disease or essential tremor. Significant tremor alleviation in the target hand ensued (*t*(12) = 7.21, *p* < 0.001, two-tailed), which was strongly associated with the functional reorganization of the brain’s hand region with the cerebellum (*r* = 0.91, *p* *<* 0.001, one*-*tailed). This reorganization potentially reflected a process of normalization, as there was a trend of increase in similarity between the hand cerebellar connectivity of the patients and that of a matched, healthy control group (*n* = 48) after treatment. Control regions in the ventral attention, dorsal attention, default, and frontoparietal networks, in comparison, exhibited no association with tremor alleviation and no normalization. More broadly, changes in functional connectivity were observed in regions belonging to the motor, limbic, visual, and dorsal attention networks, largely overlapping with regions connected to the lesion targets. Our results indicate that MRgFUS is a highly efficient treatment for tremor, and that lesioning the VIM may result in the reorganization of the cerebello-thalamo-cortical tremor network.

## Introduction

Tremor is a prominent symptom in movement disorders. It is usually observed at rest in tremor-dominant Parkinson’s disease (TDPD), while essential tremor (ET) typically involves kinetic or postural tremor^1^. The first line of treatment for tremor is pharmacological. However, medications offer limited effectiveness, especially as the disorders progress, and can cause adverse effects. As a result, about one third of patients discontinue their medication^2^. In patients for whom tremor causes a severe reduction in quality of life, invasive procedures are explored. These include deep brain stimulation (DBS) and radiofrequency surgery. The ventral intermediate nucleus (VIM) of the thalamus is currently the standard of care target for ET and a common target for TDPD^3^. The VIM is a motor thalamic nucleus which acts as a relay between the motor cortex and cerebellum^4^, and is part of a cerebello-thalamo-cortical tremor network^5^.

The most common surgical procedure for tremor alleviation is DBS. Electrodes are implanted in the brain and stimulation is delivered through a neurostimulator. The main advantages of DBS are that it is reversible (stimulation can be stopped at any time) and its programming can be adjusted and tailored to each individual to optimize symptom control. Although effective, DBS comes with important risks, such as infections, intracerebral hemorrhage, and hardware-related complications^6–9^. Another major drawback is the necessity for frequent follow-ups. In China, where much of the population lives in rural areas, this renders the procedure prohibitive for many patients. Similarly, radiofrequency thalamotomy is very effective at reducing tremor but involves the same surgery-related risks as DBS, and often comes with persistent adverse effects that remain at long-term follow-up^10^.

Ultrasound procedures have gained traction in the last decade due to technical advances in phased-array transducers^11^, leading to ultrasound devices being approved for use in movement disorders by federal regulatory agencies around the world. High-intensity Magnetic Resonance-guided Focused Ultrasound (MRgFUS) works by focusing ultrasound beams on a single target, where the concentration of energy can be harnessed to lesion brain tissue. This technology allows to bypass the skull and to correct for distortions in focal energy deposition^12^. MRgFUS has several advantages: it is minimally invasive, preserves tissue surrounding the target, and grants millimeter precision targeting. Another key advantage is that it allows to refine the location of the target before the lesion is made. Thermal energy below what is needed to lesion tissue can be deposited at the initial target site. Biofeedback in the form of alleviation of tremor can then be used to confirm the target or to adjust its location, while monitoring the patient for adverse effects. Once the target site is finalized, sonications are performed until a certain temperature is reached, leading to irreversible thermal coagulation^11^.

Several MRgFUS targets have been investigated for movement disorders. In PD, the target depends on the symptoms that need to be controlled. VIM ablation is efficacious for tremor control, but typically does not affect other motor symptoms such as rigidity and bradykinesia. Subthalamic nucleus (STN) ablation, however, targets all of those symptoms^13–15^. The globus pallidus interna (GPi) is another established MRgFUS target for PD. Its more lateral position compared to the STN or VIM can make it more challenging to focus ultrasound beams and generate a lesion^16^. Nevertheless, recent studies have found GPi MRgFUS to successfully reduce cardinal symptoms of PD, including tremor, rigidity, and bradykinesia^16,17^. Pallidothalamic tract (PTT) ablation was also investigated, and likewise resulted in improvements in motor sympoms^18–20^. In TDPD, the VIM has been used as a target almost exclusively. A recent study^21^, however, tested dual-target MRgFUS in three patients, targeting the VIM and PTT, which resulted in attenuation of tremor, rigidity, and bradykinesia. In ET, as mentioned above, targeting the VIM is the standard of care. However, another target, the cerebellothalamic tract (CTT), located in the posterior subthalamic area (PSA), has shown promise. Two studies investigated unilateral and bilateral CTT MRgFUS, which yielded excellent tremor control^22,23^. The same was found in two other studies that performed multiple-target MRgFUS, which included the CTT in addition to the VIM or other thalamic targets^19,24^.

In the current nonrandomized controlled trial, we applied MRgFUS in a sample of TDPD and ET patients with substantial hand tremor to investigate the brain’s functional changes following precise VIM lesioning, particularly the changes in cortical and cerebellar functional connectivity (FC) networks involved in hand movement. We chose the VIM as a target since the current study represents one of the first China-based MRgFUS trials for tremor treatment and as the VIM has the most evidence behind it out of all the targets for both TDPD and ET^25–31^. Patients underwent MRI scanning and tremor symptom assessment at baseline (one or two days before the intervention), one day after the intervention, as well as one month, six months, and 12 months later.

## Results

### Participants

**Fig. 1:**
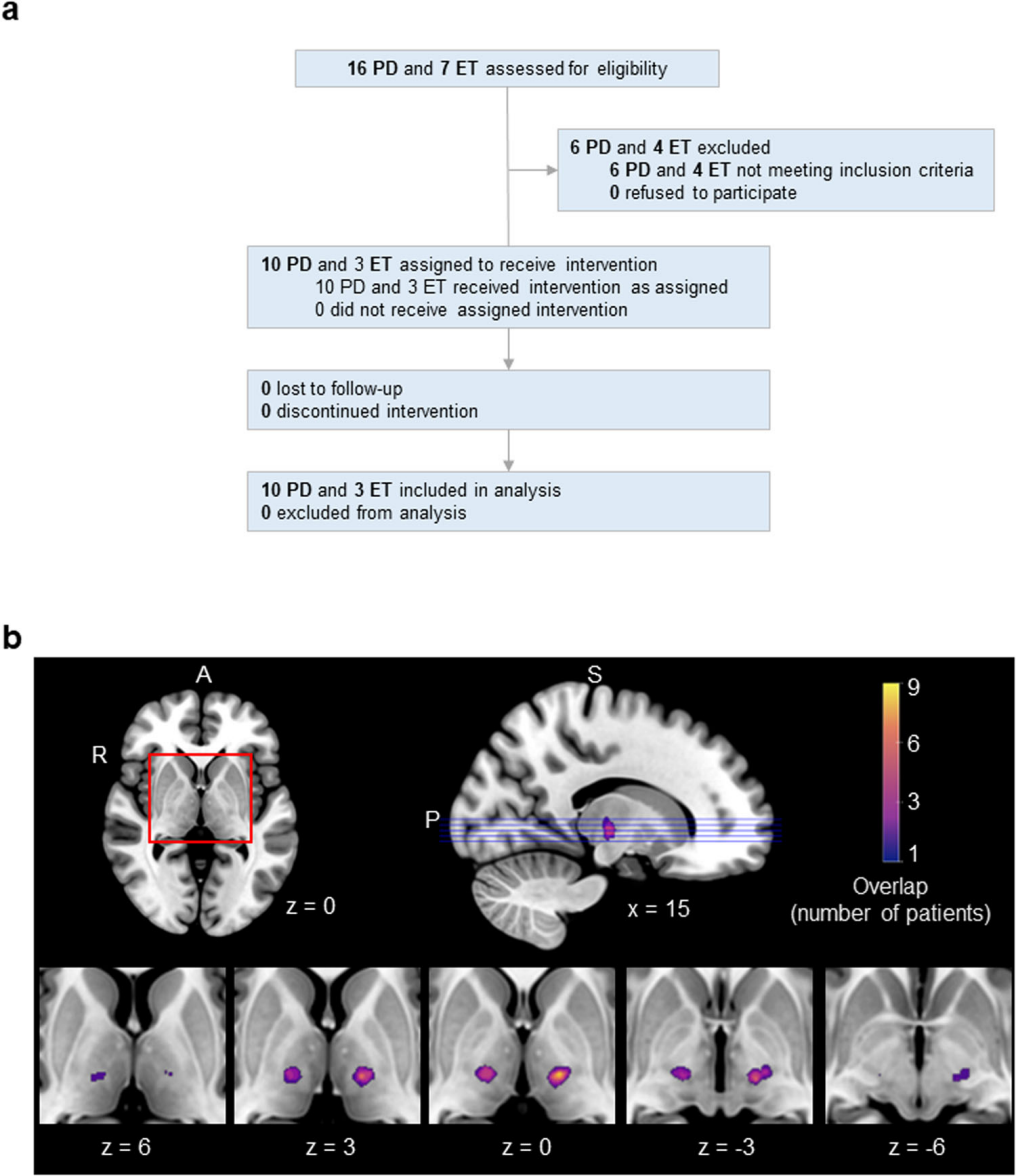
Study flow diagram and lesion sites in 13 patients with Parkinson’s disease and essential tremor. **a** Flow diagram of the intervention study. In addition to the patients, 50 control participants were enrolled. Two were excluded because they did not meet study criteria, and 48 were retained. **b** The extent of lesion overlap across patients is color-coded according to the color bar, which depicts a range from one patient to the maximum overlap found—nine patients. Lesions were localized to the left hemisphere in nine patients, and to the right hemisphere in four patients. Axial slices are shown along with a midsagittal view of the ICBM52 MNI brain. The numbers below the axial slices indicate MNI z coordinates. The position of the axial slices is shown on the midsagittal view (purple lines). The lesion location is highly similar across patients, with 9/9 patients showing overlap in the left hemisphere and 4/4 patients showing overlap in the right hemisphere.

Thirteen patients with uncontrolled tremor underwent the intervention (average age: 55.00 ± 7.58; 11 men, 2 women) (see Fig. 1a for the study’s flow diagram). Their demographics and MRgFUS characteristics are listed in Table 1. They were recruited between February and April 2019; initial testing took place between February and September 2019, and the one-month follow-up took place between March and October 2020. A control group of 48 healthy participants was included in the study (average age: 55.54 ± 6.56; 22 women, 26 men).

**Table 1:** Demographics and MRgFUS information.

	*n*	Average (SD)
Diagnosis	10 TDPD; 3 ET	–
Sex	2F; 8M	–
Target hand	6R; 4L	–
Age	–	55.00 (7.29)
Illness duration	–	4.92 (1.59)
Lesion volume (cc)	–	0.16 (0.07)
MRgFUS		
No. of sonications	–	11.15 (1.29)
Max. temp. (°C)	–	63.38 (2.50)

### VIM lesioning through MRgFUS alleviates tremor

**Fig. 2:**
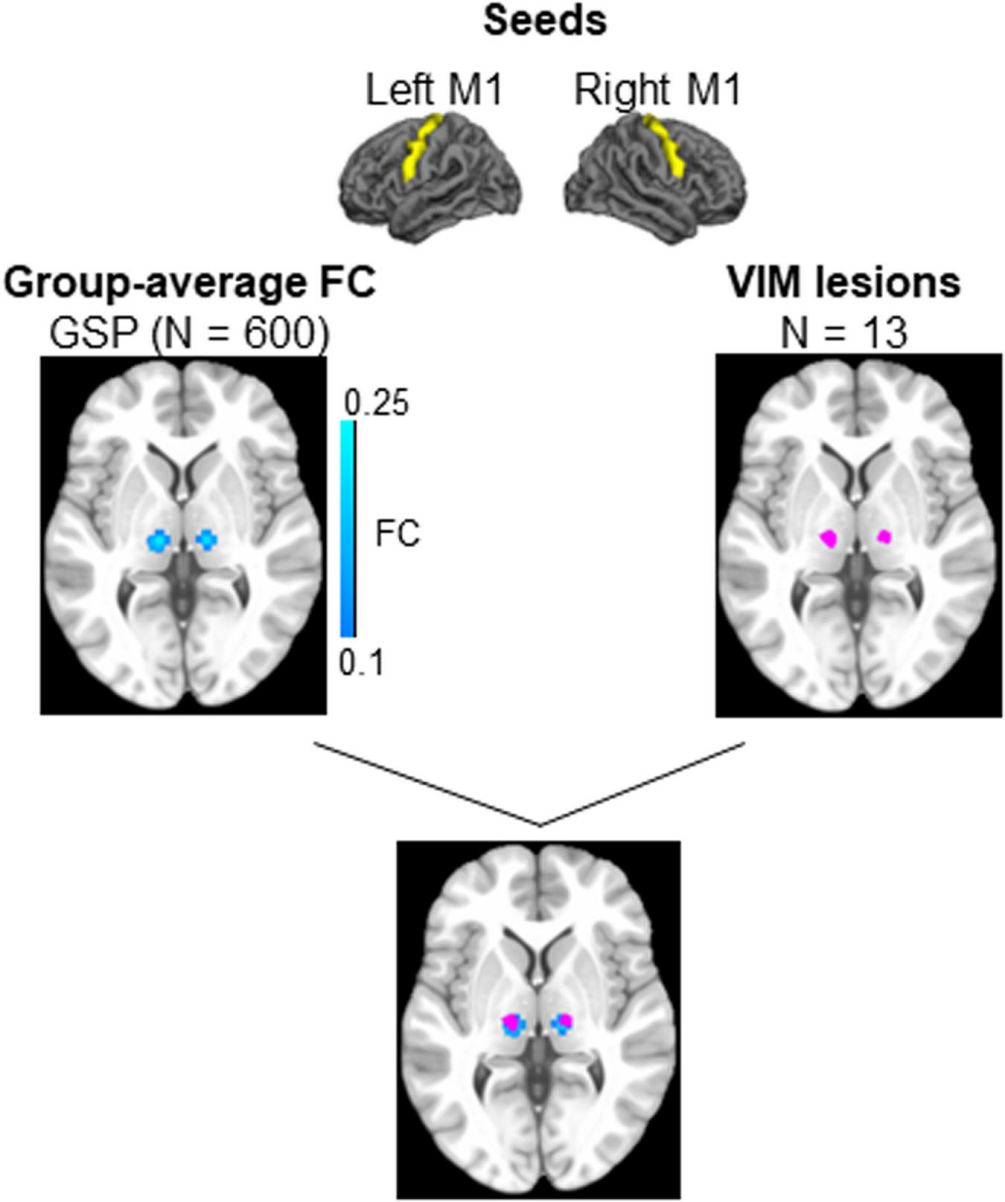
The VIM lesion sites correspond with the thalamus’s motor regions. Using the GSP dataset (*N* = 600), we defined the thalamus’s motor regions using the primary motor cortices as seeds (top). We calculated the functional connectivity of these seeds within the thalamus (middle, left) and compared the resulting thalamic regions of interest with the lesion sites in the 13 patients (middle, right). There is substantial overlap between the two (bottom), indicating that the VIM lesion sites correspond with the thalamus’s motor regions.

Lesions were traced on T2-weighted MRI scans collected the day after the intervention. Lesions were highly localized, as shown in Fig. 1b, where all patients showed overlap. A normative map of the primary motor cortex connectivity with the thalamus shows that the lesion locations correspond with the motor part of the thalamus (Fig. 2).

**Fig. 3:**
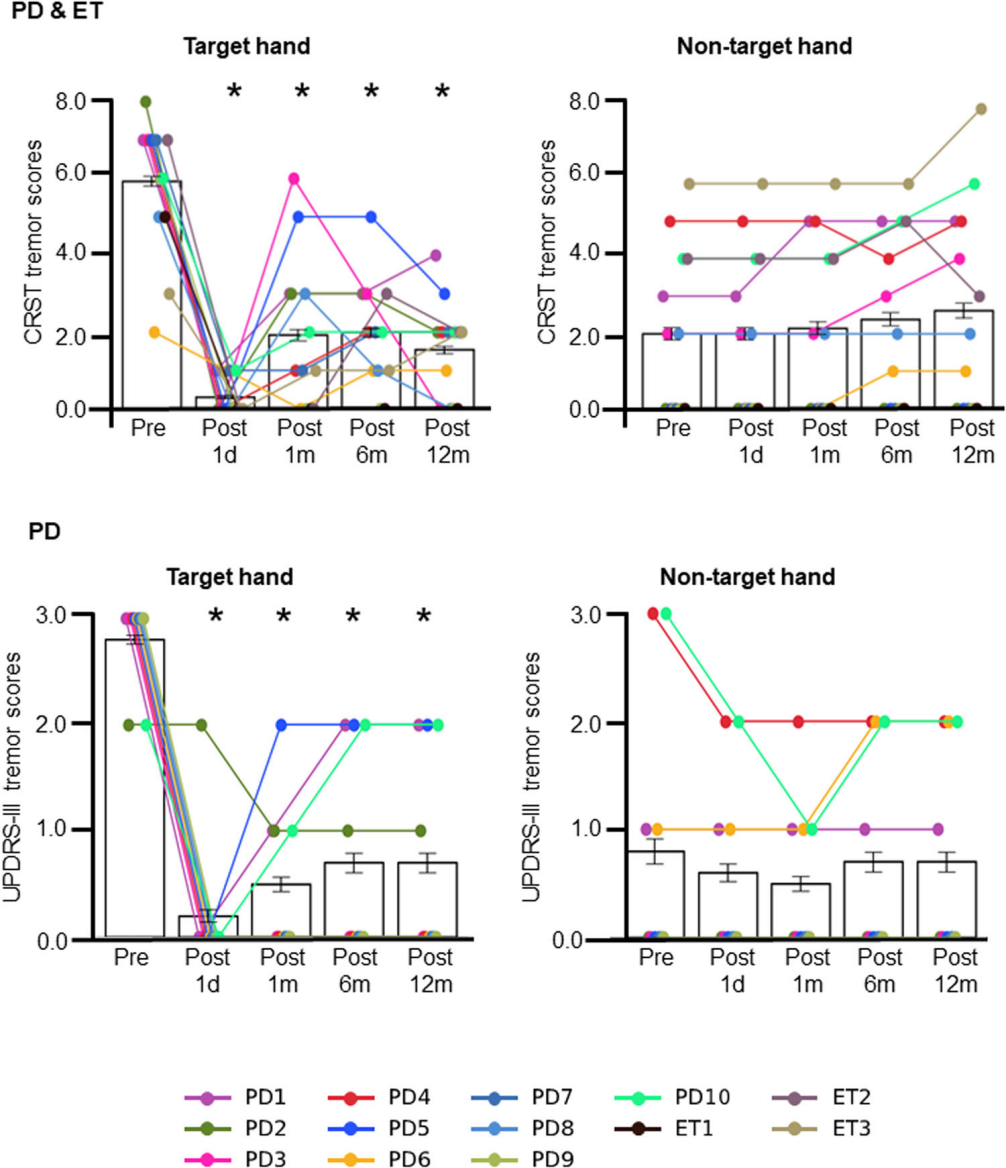
MRgFUS successfully alleviated tremor in the target hand. CRST tremor scores are shown for all 13 patients (TDPD and ET; top), for the target hand (left) and non-target hand (right), at each time point. There was a significant decrease in average target hand CRST tremor scores following the intervention (*F*(4,48) = 40.07, *p* < 0.001; top left). Scores decreased from baseline at all time points (all *p*’s < 0.001, Sidak correction; see Table 2). There was also a decrease in UPDRS-III target hand tremor scores after the procedure (*F*(4,36) = 26.70, *p* < 0.001; bottom left), which was significant at all time points (all *p*’s < 0.01, Sidak correction; see Table 2). Meanwhile, as expected, depending on the scale used, tremor in the non-target hand either increased following the intervention (CRST: *F*(4,48) = 3.01, *p* = 0.027, although post-hoc tests showed no significant different from baseline at any time point; top right) or did not change (UPDRS-III: *F*(4,36) = 1.35, *p* > 0.05; bottom right). Error bars represent standard errors of the mean. Each line connects datapoints from a given participant.

To assess tremor, all patients were administered the Fahn-Tolosa-Marin Clinical Rating Scale for Tremor (CRST)^32^. Patient with TDPD were additionally given the Unified Parkinson’s Disease Rating Scale (UPDRS)^33^ (see Methods for more details on tremor scores). All patients demonstrated an alleviation of tremor in their target hand following the MRgFUS intervention, whether assessed with the CRST or the UPDRS (Fig. 3; Table 2). A repeated measures analysis of variance (ANOVA) revealed a significant effect of Time (defined as the five time points: baseline as well as 1 day, 1 month, 6 months, and 12 months after the intervention) on CRST target hand tremor scores (*F*(4,48) = 40.07, *p* < 0.001). Post-hoc tests showed that tremor was significantly decreased from baseline at every post-intervention time point (all *p*’s < 0.001, Sidak correction; see Table 2 for detailed statistics). There was also a significant effect of Time on UPDRS-III target hand tremor scores (*F*(4,36) = 26.70, *p* < 0.001), and post-hoc tests again showed significant decreases at each time point compared to baseline (1 day and 1 month: *p*’s < 0.001, 6 months and 12 months: *p*’s = 0.004, Sidak correction; see Table 2). Conversely, CRST tremor in the non-target hand exhibited a significant increase over Time (*F*(4,48) = 3.01, *p* = 0.027). However, post-hoc tests indicated that none of the time points showed a significant difference from baseline (all *p*’s > 0.05, Sidak correction; see Table 2). No significant difference over Time was found for UPDRS-III non-target hand tremor scores (*F*(4,36) = 1.35, *p* > 0.05). Tremor alleviation was not related to lesion size (CRST: *r* = −0.20, *p* = 0.53, two-tailed; UPDRS: *r* = 0.18, *p* = 0.65, two-tailed).

**Table 2:** Tremor and mood scores.

				Paired *t*-test		
	*n*	Average score (SD)	Mean difference from baseline	*t*	*p*	95% CI
CRST tremor						
Target hand						
Baseline	13	5.92 (1.69)	–	–	–	–
1 day	13	0.31 (0.46)	5.62	–	<0.001	3.82, 7.41
1 month	13	1.92 (1.90)	4.00	–	<0.001	2.11, 5.90
6 months	13	2.00 (1.36)	3.92	–	<0.001	2.56, 5.29
12 months	13	1.54 (1.22)	4.38	–	<0.001	2.68, 6.09
Non-target hand						
Baseline	13	2.00 (2.11)	–	–	–	–
1 day	13	2.00 (2.11)	0.00	–	–	0.00, 0.00
1 month	13	2.15 (2.25)	−0.15	–	0.98	−0.68, 0.37
6 months	13	2.38 (2.27)	−0.38	–	0.64	−1.11, 0.34
12 months	13	2.62 (2.65)	−0.62	–	0.43	−1.60, 0.37
UPDRS-III tremor						
Target hand						
Baseline	10	2.80 (0.40)	–	–	–	–
1 day	10	0.20 (0.60)	2.60	–	<0.001	1.47, 3.72
1 month	10	0.50 (0.67)	2.30	–	<0.001	1.20, 3.40
6 months	10	0.70 (0.90)	2.10	–	0.004	0.71, 3.49
12 months	10	0.70 (0.90)	2.10	–	0.004	0.71, 3.49
Non-target hand						
Baseline	10	0.80 (1.17)	–	–	–	–
1 day	10	0.60 (0.80)	0.20	–	0.84	−0.29, 0.69
1 month	10	0.50 (0.67)	0.30	–	0.88	−0.48, 1.08
6 months	10	0.70 (0.90)	0.10	–	1.00	−0.56, 0.76
12 months	10	0.70 (0.90)	0.10	–	1.00	−0.56, 0.76
Total CRST Part A						
Baseline	13	15.38 (6.75)	–	–	–	–
1 day	13	5.23 (4.26)	10.15	–	–	–
1 month	13	8.92 (4.89)	6.46	–	–	–
6 months	13	9.00 (4.76)	6.38	–	–	–
12 months	13	8.54 (5.15)	6.85	–	–	–
Total UPDRS-III						
Baseline	10	29.70 (8.60)	–	–	–	–
1 day	10	17.80 (6.87)	11.90	–	–	–
1 month	10	21.30 (6.34)	8.40	–	–	–
6 months	10	26.00 (6.45)	3.70	–	–	–
12 months	10	29.10 (4.91)	0.60	–	–	–
BDI						
Baseline	13/10	6.85 (4.19)/8.20 (3.84)	–	–	–	–
1 day	13	5.31 (3.52)	1.54	–	–	–
1 month	13	4.85 (3.42)	2.00	2.55	0.03	0.29, 3.71
6 months	10	6.80 (2.44)	1.40	–	–	–
12 months	10	7.00 (2.90)	1.20	2.17	0.058	−0.05, 2.45

### Impact on mood and adverse effects

We sought to determine whether the MRgFUS intervention was associated with any changes in depressive symptoms, as assessed with the Beck Depression Inventory (BDI)^34^. ET patients were not administered the BDI at long-term follow-ups (1 and 12 months), therefore we investigated changes both at one month after the procedure (*n* = 13) and at 12 months (*n* = 10). Compared to baseline, paired *t*-tests showed that there was a significant decrease in BDI scores at 1 month (*t*(12) = 2.55, *p* = 0.03, two-tailed; mean difference = 2.00, two-sided 95% CI [0.29, 3.71]), and a marginally significant decrease at 12 months (*t*(9) = 2.17, *p* = 0.058, two-tailed; mean difference = 1.20, two-sided 95% CI [−0.05, 2.45]) (Table 2).

Adverse effects one month after treatment included gait disturbance in three patients (23%), and paresthesia or numbness in five patients (37%). At 6 months, one PD patient presented with inflexible movement and slow reaction in the target hand, and another exhibited slight limb shaking in the leg on the treated side. By 12 months, all of the adverse effects had resolved.

### Impact on the functional organization of the brain

**Fig. 4:**
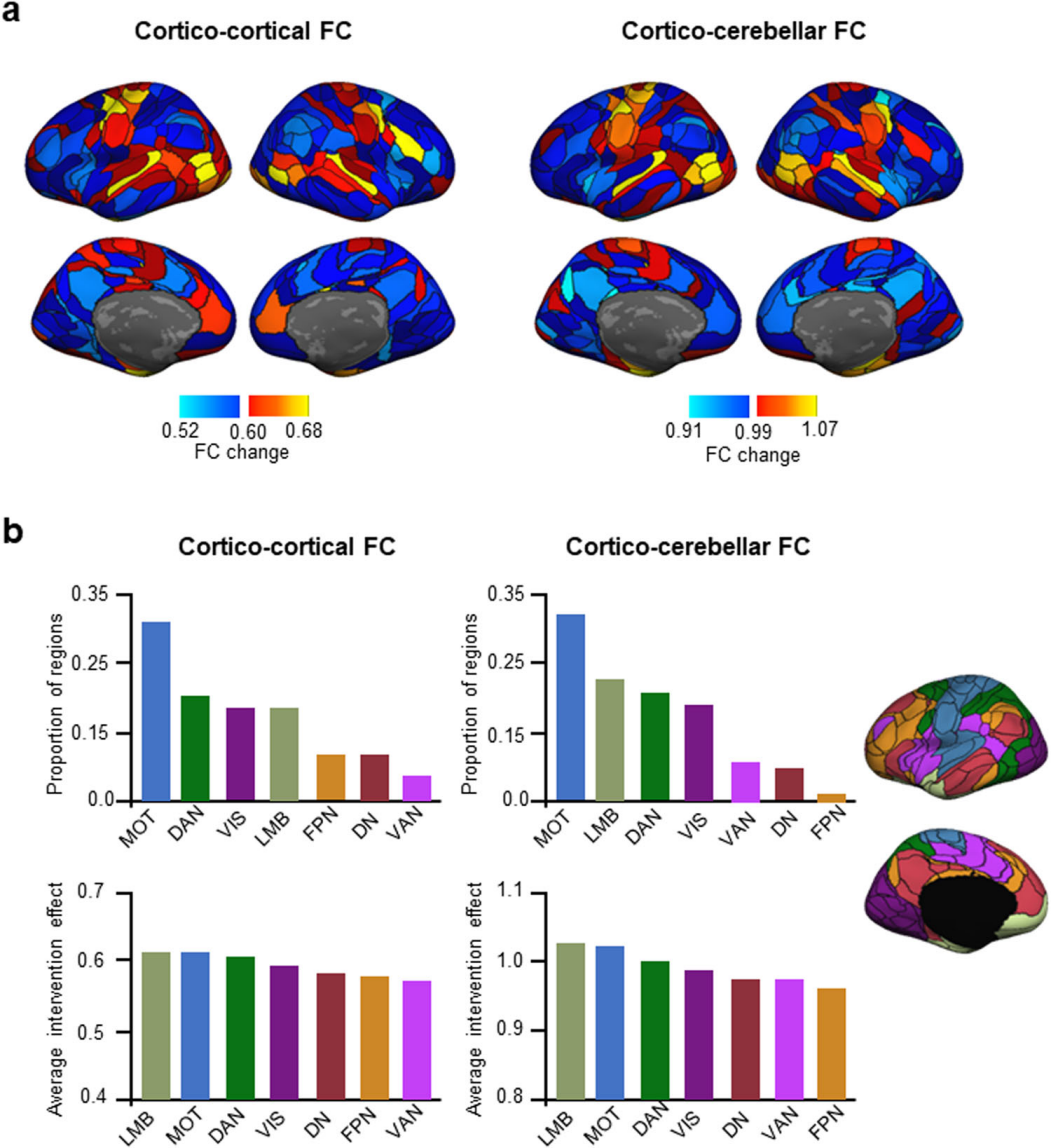
The motor, limbic, and visual large-scale functional networks are most affected by the VIM lesion. **a** We investigated cortico-cortical (left) and cortico-cerebellar (right) functional connectivity to determine which regions exhibited an intervention effect, i.e., regions whose functional connectivity substantially changed from pre- to post-intervention. Regions in warm colors demonstrated an above-average intervention effect. The color bar indicates FC change, which was calculated as the dissimilarity between pre- and post-FC profiles while controlling for intra-individual variability (see Methods), with the middle value representing the mean. **b** Cortical regions were divided into the seven canonical large-scale functional networks (depicted on the right). The networks with the highest proportion of regions exhibiting an intervention effect at the level of cortico-cortical FC were the MOT, DAN, VIS, and LMB networks (top left panel). The average intervention effect of each network was calculated. The networks that demonstrated the largest average intervention effect in terms of their cortico-cortical FC were the LMB, MOT, DAN, and VIS networks (bottom left panel). In terms of cortico-cerebellar FC, the networks with the highest proportion of regions demonstrating an intervention effect were the MOT, LMB, DAN, and VIS networks (top right panel). The highest average intervention effect was found in the LMB, MOT, DAN, and VIS networks (bottom right panel). MOT sensorimotor network, DN default network, LMB limbic network, VIS visual network, VAN ventral attention network, DAN dorsal attention network, FPN frontoparietal network.

To explore the effects of MRgFUS on the fine and coarse functional organization of the brain, we parcellated the cortex into fine-grained functional networks as well as large-scale functional networks, and calculated each network’s change in FC from pre- to post-treatment while taking into account normal within-individual variability in FC (see Methods). The large-scale networks correspond to those described by Yeo et al.^35^ and include: the sensorimotor network (MOT), default network (DN), limbic network (LMB), visual network (VIS), ventral attention network (VAN), dorsal attention network (DAN), and frontoparietal network (FPN). We did not expect to find a statistically significant effect as the lesions were very small (average = 163.1 mm^3^; Table 1) and due to the limited sample size, thus we deemed regions to have exhibited a change in FC if they exceeded the mean of all regions’ FC change. The regions most affected by the intervention were mainly located along the sensorimotor strip, for both cortico-cortical FC and cortico-cerebellar FC (Fig. 4a). In terms of large-scale networks, these regions mainly belonged to the MOT, DAN, LMB, and VIS networks, whether we considered their FC with the cortex or with the cerebellum (Fig. 4b, top). Concordantly, the large-scale functional networks that demonstrated the largest average FC change were the LMB, MOT, DAN, and VIS (Fig. 4b, bottom), although the intervention effect was similar across all networks.

**Fig. 5:**
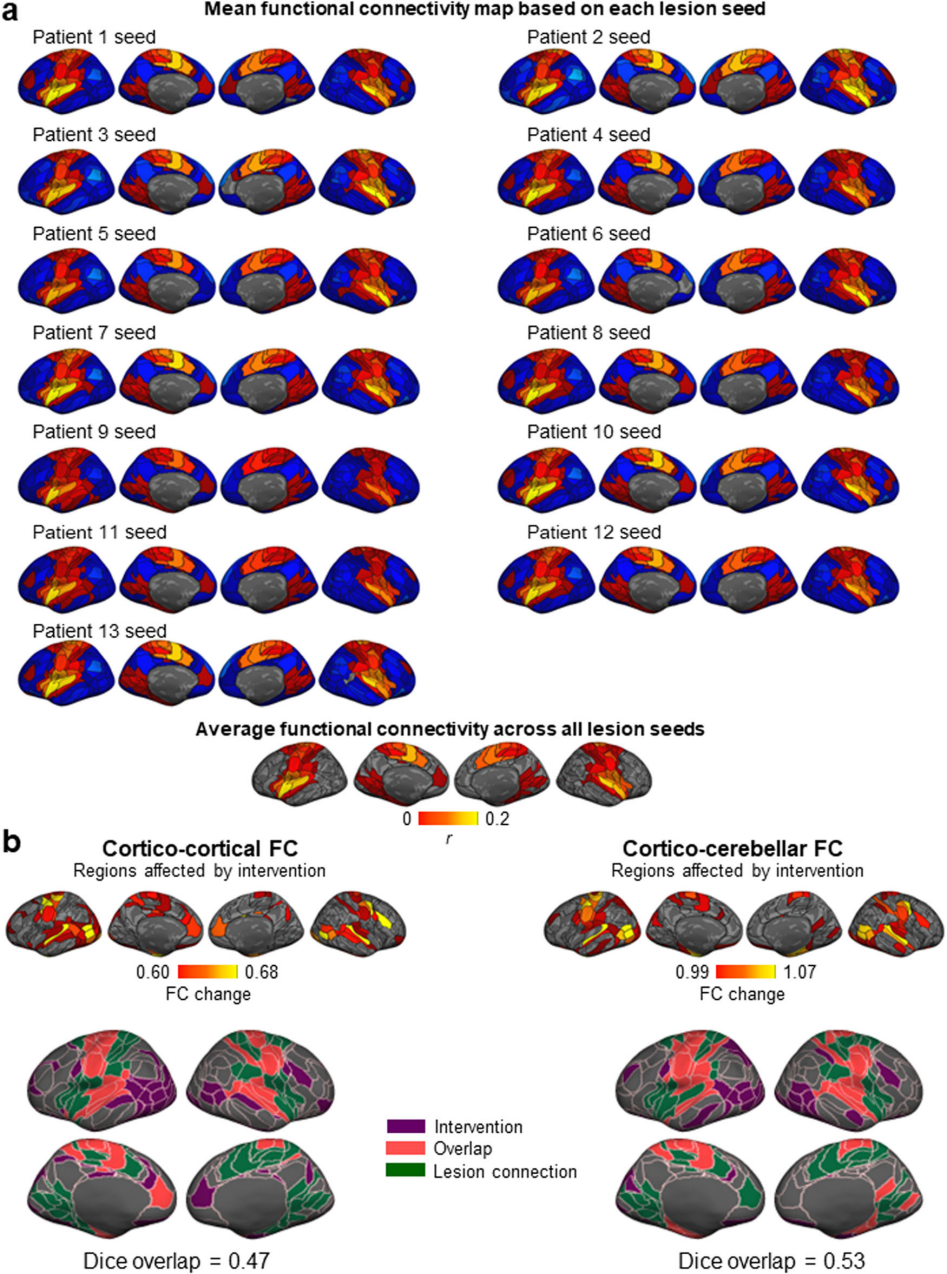
Regions functionally connected to the lesion sites exhibited a change in their functional connectivity following the intervention. **a** Functional connectivity of the lesion site. The lesion site of each patient was used as a seed to generate a functional connectivity map, based on data from the control group (*N* = 48). The resulting FC maps are shown for each lesion site. The average FC map, across all 13 seeds, is shown at the bottom. The color bar represents FC strength (Pearson *r*). **b** The cortical maps depict the cortical regions whose functional connectivity substantially changed from pre- to post-intervention, according to their cortico-cortical (middle left panel) and cortico-cerebellar (middle right panel) functional connectivity. The color bar indicates FC change, and only values above the mean are shown. We then calculated the extent of overlap between the regions connected to the lesion sites and the regions that demonstrated an intervention effect. The overlap was Dice = 0.47 for cortico-cortical connections (bottom left panel), and Dice = 0.53 for cortico-cerebellar connections (bottom right panel). These substantial overlaps indicate that the functional reorganization that occurred after the intervention mainly affected the regions that are functionally connected to the lesion sites.

Next, we sought to determine whether the regions that were most affected by the intervention were the same regions that are functionally connected to the lesion target. We generated FC maps, using the control group, of all 13 lesion sites, and averaged them to produce a normative FC map of the approximate location of the VIM (Fig. 5a). Whether considering cortico-cortical FC or cortico-cerebellar FC, there was substantial overlap between the two sets of regions (cortico-cortical FC: Dice = 0.47; cortico-cerebellar FC: Dice = 0.53; Fig. 5b), indicating that the regions affected by the MRgFUS intervention were functionally connected with the lesion target. This overlap mostly consisted of regions from the MOT network.

### Association between FC change in hand region and tremor alleviation

**Fig. 6:**
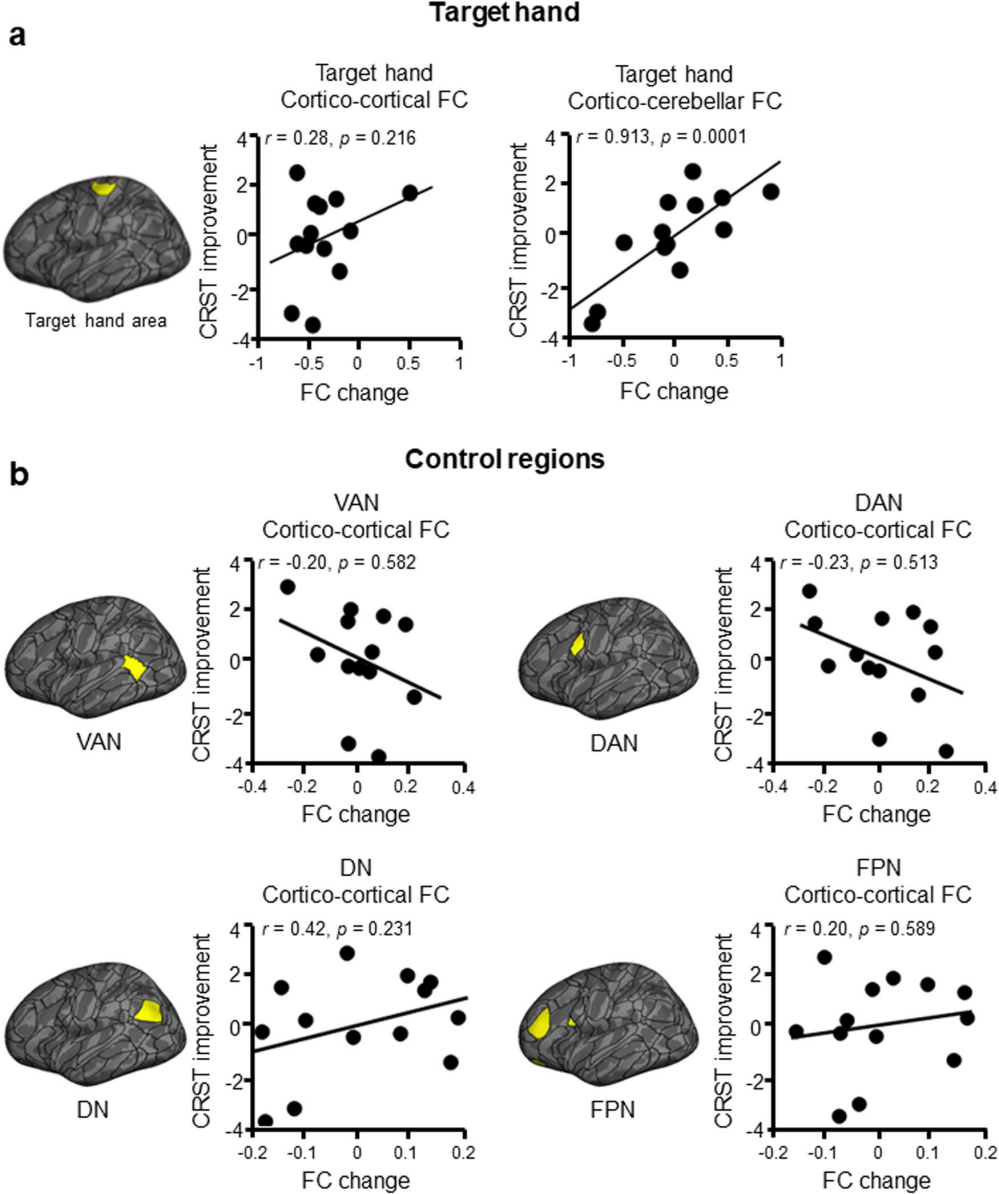
Association between FC change and tremor alleviation. **a** Partial Spearman correlations, covaried with age and sex, between FC change and tremor alleviation demonstrated a non-significant, positive relationship for the cortical target hand region’s cortical FC (*r* = 0.28, *p* = 0.22, one-tailed; left), and a significant, positive relationship for its cerebellar FC (*r* = 0.91, *p* < 0.001, one-tailed; right). **b** The same analysis, performed in four control regions selected at random, showed non-significant relationships for the cortical FC of the VAN control region (r = −0.20, *p* = 0.58, two-tailed; right), DAN control region (*r* = −0.23, *p* = 0.51, two-tailed), DN control region (*r* = 0.42, *p* = 0.23, two-tailed), and FPN control region (*r* = 0.20, *p* = 0.59, two-tailed). DN default network, VAN ventral attention network, DAN dorsal attention network, FPN frontoparietal network.

The brain’s hand regions were of specific interest in this study as the goal of the MRgFUS intervention was to alleviate hand tremor. Seeing as there were substantial amelioration in hand tremor symptoms, we investigated the association between tremor alleviation in the target hand and FC change in the brain’s target hand region. We investigated both cortical and cerebellar connectivity in these analyses as the VIM and motor cortex have important connections with these structures. The cortical target hand region demonstrated a non-significant relationship in terms of its cortical FC change (*r* = 0.28, *p* = 0.22, one-tailed; Fig. 6a, left), and a strong, significant relationship in terms of its cerebellar FC change (*r* = 0.91, *p* < 0.001, one-tailed; Fig. 6a, right). Four randomly chosen regions, which belonged to the VAN, DAN, DN, and FPN, and which served as control, demonstrated no significant relationships (VAN: *r* = −0.20, *p* = 0.58, two-tailed; DAN: *r* = −0.23, *p* = 0.51, two-tailed; DN: *r* = 0.42, *p* = 0.23, two-tailed; FPN: *r* = 0.20, *p* = 0.59, two-tailed; Fig. 6b).

### Similarity with normative FC

**Fig. 7:**
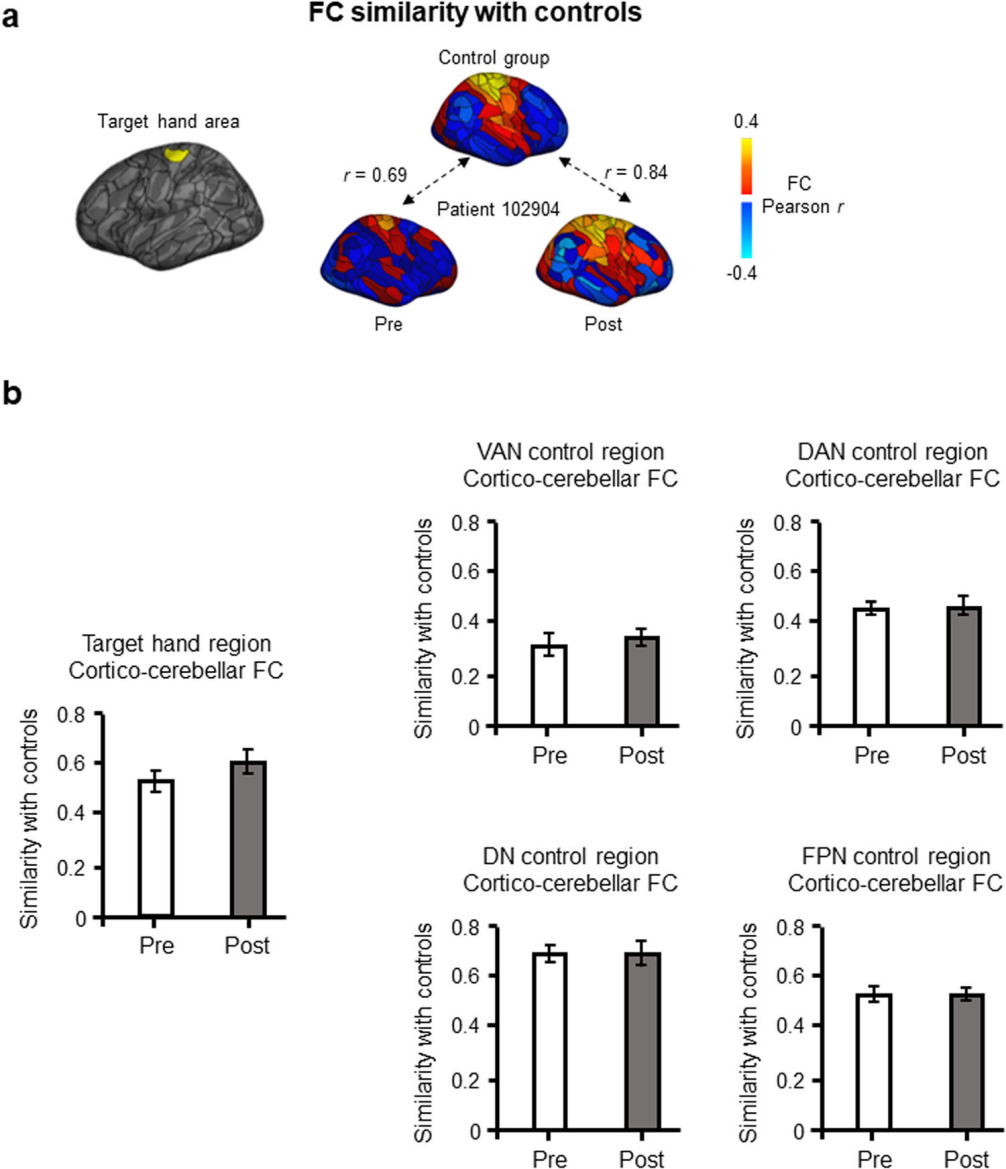
Cortico-cerebellar FC similarity before and after the intervention. **a** The left hemisphere hand region is highlighted on a cortical map (left). For each patient, we calculated the similarity in functional connectivity patterns between their cortical target hand region and the control group’s left hemisphere hand region, before and after the MRgFUS intervention. An example is shown for a randomly chosen patient, whereby the FC similarity is *r* = 0.69 before the intervention, and *r* = 0.84 after the intervention. The increased similarity from pre- to post-intervention indicates a normalization of the target hand region’s functional organization following VIM lesioning. **b** We averaged the FC similarity for the target hand region across patients at each time point. There was a non-significant trend of increase in cortico-cerebellar FC similarity from pre- to post-intervention (*t*(12) = −1.18, *p* = 0.13, one-tailed; mean difference = −0.07, one-sided 95% CI [−0.19, 0.04]). The four control regions, in comparison, showed either comparatively smaller or no change in FC similarity, and were all non-significant (VAN: *t*(12) = −0.71, *p* = 0.49, two-tailed; mean difference = −0.03, two-sided 95% CI [−0.11, 0.06]); DAN: *t*(12) = −0.33, *p* = 0.74, two-tailed; mean difference = −0.01, two-sided 95% CI [−0.08, 0.06]; DN: *t*(12) = 0.03, *p* = 0.96, two-tailed; mean difference = 0.00, two-sided 95% CI [−0.06, 0.06]; FPN: *t*(12) = 0.04, *p* = 0.97, two-tailed; mean difference = 0.00, two-sided 95% CI [−0.07, 0.07]). DN default network, VAN ventral attention network, DAN dorsal attention network, FPN frontoparietal network.

To probe the potential mechanism behind the hand region’s FC change and its association with tremor alleviation, we investigated whether the cortical target hand region’s connectivity with the cerebellum was more similar to the healthy control group’s following the intervention relative to immediately prior (see Fig. 7a for an example; see Methods for details). There was a non-significant, slight trend of increase in similarity of the target hand region’s cerebellar FC (*t*(12) = −1.18, *p* = 0.13, one-tailed; mean difference = −0.07, one-sided 95% CI [−0.19, 0.04]; Fig. 7b, top). The four control regions’ cerebellar FC similarity, in comparison, did not change (VAN: *t*(12) = −0.71, *p* = 0.49, two-tailed; mean difference = −0.03, two-sided 95% CI [−0.11, 0.06]; DAN: *t*(12) = −0.33, *p* = 0.74, two-tailed; mean difference = −0.01, two-sided 95% CI [−0.08, 0.06]; DN: *t*(12) = 0.03, *p* = 0.96, two-tailed; mean difference = 0.00, two-sided 95% CI [−0.06,0.06]; FPN: *t*(12) = 0.04, *p* = 0.97, two-tailed; mean difference = 0.00, two-sided 95% CI [−0.07, 0.07]; Fig. 7b, bottom).

## Discussion

The present study showed high efficacy of MRgFUS in alleviating tremor; all patients exhibited decreased tremor in the treated hand. When considering all 13 patients, there was 68% improvement on average at 1 month, as assessed with the CRST subitems for rest, posture, and action/intention tremor. At 1 year, the improvement was 74% (Table 2). The 10 patients with TDPD experienced an improvement averaging 82% at the one-month follow-up, as assessed with the rest tremor subitem of the UPDRS. One year following the intervention, the improvement was 75% (Table 2). Global motor scores improved by 41% in all patients (total CRST Part A scores) at 1 month and 44% at 1 year (Table 2). These alleviations are comparable to those observed in prior MRgFUS studies in which the VIM was lesioned, where improvements in target hand/side tremor ranging 47–90% were observed^25,26,29–31^, and global motor scores improved 34.1–49.7%^27,28^. In addition, the beneficial effects on tremor were sustained one year after treatment, indicating that the clinical benefits were stable and long-lasting.

Lesion volume averaged 163.1 mm^3^ the day after the MRgFUS procedure, which is comparable to those previously reported, e.g. Schlesinger and colleagues^27^= 231 mm^3^ on day 1; Lipsman et al.^31^ = 6.3 mm in diameter on day 1 (sphere: 250 mm^3^); Fasano et al.^26^ = 6–8 mm in diameter on day 1 (sphere: 216–512 mm^3^); Zaroor et al.^28^ = 6.8 mm in diameter on day 1 (sphere: 314 mm^3^).

In terms of adverse effects, patients experienced gait disturbances (3/13 patients: 23%) and paresthesia (5/13 patients: 38%) 1 month after the procedure, as well as inflexibility and slowness of movement in the target hand (1/13 patients: 8%) and slight shaking of the leg (1/13 patients: 8%) 6 months after treatment. None of these adverse effects persisted beyond the 12-month follow-up. Similar studies of unilateral VIM MRgFUS with long-term follow-up also reported adverse effects that either resolved or persisted in a small number of patients 12 months after treatment^25,27,28,30,36–45^.

To investigate the functional reorganization of the brain, the cortico-cortical and cortico-cerebellar FC of individual-defined cortical regions was compared before and after the MRgFUS intervention. As expected, lesioning the VIM did not cause a significant change in whole-brain FC, which we postulate is due to the small lesion sizes as well as the small sample. Nonetheless, the areas that exhibited a subthreshold change included the primary motor and somatosensory cortex, supplementary motor cortex, premotor cortex, superior temporal cortex, visual areas in the occipital cortex, and the anterior cingulate cortex. There was considerable overlap between these regions and the regions that are functionally connected to the VIM. This indicates that VIM lesioning is likely the cause of the FC change observed in these regions.

These findings are in large agreement with studies that investigated FC and the VIM. Zhang et al.^46^ found that the VIM is functionally connected to the primary motor cortex, premotor cortex, supplementary motor area, and temporal lobe, among other regions. Al-Fatly et al.^47^ identified multiple regions whose FC with deep-brain stimulation targets in and around the VIM was related with positive motor outcomes. These included the primary motor and sensory cortex, secondary motor cortex, premotor cortex, supplementary motor area, visual cortex, and superior temporal gyrus. Thus, the regions that exhibited a degree of change in their functional organization are regions known to be part of the tremor network, and VIM lesioning likely triggered the change in their FC patterns.

When considering large-scale functional networks, the motor, limbic, dorsal attention, and visual networks exhibited the most substantial change. The change in motor network connectivity would explain the reduction in tremor experienced after VIM lesioning. The limbic network change could account for the reduction in depressive symptoms that was observed between baseline and one month after MRgFUS. The visual network has previously been implicated in disorders of tremor; visual feedback has been identified as a modulating factor for tremor severity^48^, and activity, connectivity, and grey matter in visual areas have been shown to be related to tremor severity or alleviation^47,49–51^. The dorsal attention network has also been implicated, as its FC was found to have undergone change after radiosurgery of the VIM^50^, and the FC of regions that are part of the dorsal attention network was found to be predictive of tremor improvement^47^.

The important alleviation in hand tremor following the intervention led us to investigate the FC of the cortical hand target region, and whether its change in FC was associated with the extent of tremor improvement. We found a strong correlation between the two, particularly when considering the hand region’s FC with the cerebellum. This finding helps explain the potential mechanism behind motor symptom improvement, whereby lesioning the VIM of the thalamus produces a change in the FC between the cortex and cerebellum, ultimately modulating tremor symptoms, and highlights an interaction between all the nodes of the cerebello-thalamo-cortical network. The strength of the correlation (*r* = 0.91) is striking, and care was taken to remove the influence of intra-individual FC variability (see Methods), thereby lending some assurance that the correlation is not overly inflated. Four randomly chosen control regions showed no such association, indicating that the significant finding is unlikely to be spurious. However, the sample size is very small, and therefore sampling bias may have nonetheless contributed to an inflated correlation. This particular association should be examined again in larger studies.

This association prompted us to investigate whether the cortical target hand region’s cerebellar FC with the cerebellum became more similar to controls’ one month after the procedure. We found a slight trend of increase in similarity, indicating normalization of the region’s FC. In comparison, the four control regions showed either numerically smaller increases or no changes at all. Normalization could help explain how the change in the target hand FC contributed to an alleviation of tremor, but its role is not confirmed in the present small-scale study.

The current study presented several limitations. The small patient sample size limited our ability to detect significant effects on whole-brain connectivity changes. However, the convergence of the regions showing the greatest change, the regions connected to the VIM, and the regions previously shown to be involved in tremor alleviation, lends some assurance that the effects observed, although non-significant, were related to the intervention and not spurious. Second, our patient sample was comprised of both TDPD and ET patients, who typically present with different tremor symptoms^1^. This likely increased the heterogeneity of the data, however the sample size benefited from combining the two groups. Third, we used a control sample of 48 participants to calculate the normative FC of the cortical hand region. While a substantial amount of data was acquired for each participant, a larger sample size would be needed to ensure that the connectivity measured is representative of a normal population. However, we deemed this approach more robust than using public datasets as the scanning parameters and demographics match those of the patients. Lastly, it was not possible in the current study to assess the generalizability of our findings; ours represents one of the first studies to use MRgFUS to treat tremor in China. Accordingly, the success of the therapy first had to be assessed, before enrolling more participants and conducting further study. Generalizability will be investigated in future studies, using independent datasets. Another crucial line of study would be to investigate alternate targets, such as the posterior subthalamic area, and whether their lesioning impacts the cerebello-thalamo-cortical tremor network differently than VIM MRgFUS.

## Methods

### Participants

In this nonrandomized controlled trial, a referred sample of 10 patients with TDPD and three patients with ET underwent MRgFUS in a hospital setting. Inclusion criteria for the patients are listed in Table 3. For this clinical trial, a statistical sample size analysis was not proposed; instead, 100 patients with medication-refractory TDPD (https://clinicaltrials.gov/ct2/show/NCT04002596) and 100 patients with ET (https://clinicaltrials.gov/ct2/show/NCT03253991) were planned to be enrolled. The current study represents one of the first in China to administer MRgFUS treatment. Therefore, before enrolling large numbers of participants, we first had to assess the success of the therapy on a small sample of patients, presented here.

**Table 3:** Inclusion criteria.

TDPD	ET
1) Tremor dominant and postural instability/gait difficulty ratio>1.15 in the medicated [ON] state. 2) ≥3 in the affected hand/arm, as measured by the medicated [ON] UPDRS question #20, or a postural/action tremor≥2 for question #21. Patients with bilateral appendicular tremor were included. 3) Substantial tremor-induced disability despite medical treatment, as determined by a Fahn-Tolosa-Marin Clinical Rating Scale for Tremor (CRST) score≥2 in any one of the items 16–23 from the Disability subsection of the CRST. 4) Stable medication for 30 days prior to study entry.	1) Inadequate response to one or two medications, per local standards. An inadequate response includes poor response to the drug, or the development of side effects. 2) Postural or intention tremor score≥2 (CRST) in the dominant hand or arm, while stable on medication. Patients with bilateral appendicular tremor were included. 3) Substantial tremor-induced disability despite medical treatment, as determined by a CRST score≥2 in any one of the items 16–23 from the Disability subsection of the CRST. 4) Stable medication for tremor for 30 days prior to study entry.

A group of 48 healthy control participants, matched to the patient group with regards to age, sex, and cognitive status, was included in the study. All participants gave written informed consent according to the Declaration of Helsinki. The study was approved by Henan Provincial People’s Hospital Institutional Review Board.

### Control group

The control group was not significantly different from the patient group in terms of age (*t*(59) = 0.26, *p* = 0.80, two-tailed; mean difference = 0.59, two-sided 95% CI [−3.70, 4.78]), sex (Fisher’s exact test, *p* = 0.06, two-tailed), or cognitive status (Mini-Mental State Examination (MMSE): t(59) = −0.74, *p* = 0.46, two-tailed; mean difference = −0.49, two-sided 95% CI [−1.80, 0.83]).

We used the GSP dataset^52^ to identify the motor part of the thalamus using FC. Resting-state fMRI data of 600 healthy young adult participants (mean age: 20.88 ± 3.01 years) were included.

### Medication information

All 10 TDPD patients were on 715 ± 330 mg (mean ± SD) of daily levodopa in the month preceding MRgFUS treatment. In the month following the procedure, patients took 516 ± 197 mg (mean ± SD) of daily levodopa. In the ET group, in the month preceding the MRgFUS procedure, the first patient took Almarl, 15 mg/day, the second patient took Pregabalin, 62 mg/day, Propranolol, 20 mg/day, and Clonazepam, 2 mg/day, and the third patient took Almarl, 20 mg/day. Following the procedure, all medication was stopped in ET patients.

### Tremor and neuropsychological assessment

All patients were administered the Fahn-Tolosa-Marin Clinical Rating Scale for Tremor **(**CRST)^32^. Because patients with TDPD mostly experience tremor at rest, and patients with ET mostly during actions, we selected the sum of the “Tremor at rest, with posture holding, and with action and intention” of the target upper extremity scores in Part A as the outcome measure. Patients with TDPD, but not ET, were administered the Unified Parkinson’s Disease Rating Scale (UPDRS)^33^. Because these patients mostly experience tremor at rest, we selected the “Rest tremor amplitude” in the target upper limb in Part III (motor examination) as the outcome measure. The Beck Depression Inventory (BDI)^34^ was administered to assess depression at each time point. However, the patients with ET were not administered the BDI at the 6-month and 12-month follow-ups. The MMSE^34^ was administered to assess cognitive status to ensure that patients and control participants were matched in this regard.

### MRgFUS procedure

MRgFUS was used to lesion the VIM contralateral to the most affected hand. Patients were awake and lying supine during the procedure. They received local anesthesia to the scalp. A stereotactic frame (Integra Radionics, Burlington, USA) was fixed to their skull. Then, the patient was positioned supine and headfirst in the MR (Discovery MR750, GE Healthcare, Milwaukee, USA)/ExAblate (ExAblate Neuro, InSightec, Haifa, Israel) Transcranial therapy table. The half-spherical helmet containing the focused ultrasound transducer elements (ExAblate Neuro, InSightec, Haifa, Israel) was positioned around the patient’s head. VIM location was estimated using T2-weighted scans (see MRI data acquisition). The approximate target location was set on the AC-PC plane, at 75% of the AC-PC line and 14 mm lateral to the AC-PC line. When there was third ventricle enlargement, the approximate target was set to 11.5 mm lateral to the third ventricle wall.

A low dose energy sonication was applied to the target location in the VIM to confirm target accuracy. These low energy sonications non-destructively warmed the target. The warming was captured by the MR thermometry, and the MR thermal images were displayed in real time and monitored by the treating physician. The neurosurgeon then verified that the warming was centered on the anatomic target. This allowed the centering of the eventual permanent thermal lesion in the planned location. The patient was examined by the treating team during and after each sonication for neurologic signs and symptoms, and evidence of tremor suppression. The treating physician was in direct communication with the patient at all times. Biofeedback ensured that a target location was found that alleviated hand tremor without eliciting other sensory changes or paresthesia in any part of the body. Lesioning was done by increasing the power and/or duration of the sonications to produce incrementally larger lesions.

For the lesion analysis, the lesions, identified using T2-weighted MRI scans collected the day after the MRgFUS intervention, were traced onto the ICBM 152 MNI template by a radiologist (L.Du) blind to patients’ clinical information, using MRIcro (www.mccauslandcenter.sc.edu/mricro/). Lesion overlap images and lesion volumes were provided using MRIcroGL (www.mccauslandcenter.sc.edu/mricrogl/).

### MRI acquisition

On each visit, one structural scan (8 min 50 s) and 5 resting-state scans (6 min 14 s each for a total duration of 31 min 10 s) were performed.

MRI data were collected using a 3 T MRI scanner (Magnetom Prisma, Siemens Healthcare, Erlangen, Germany) equipped with a 64-channel head coil. T1-weighted structural scans were acquired using a gradient echo MP2RAGE sequence with the following parameters: resolution = 1 mm isotropic, TI1 = 755 ms, TI2 = 2500 ms, TE = 3.43 ms, TR = 5000 ms, flip1 = 4°, flip2 = 5°, bandwidth=240 Hz/pix, echo spacing=7.1 ms, matrix = 256 × 256, 208 slices, acceleration factor of 3 (32 reference lines) in the primary phase encoding direction and online GRAPPA image reconstruction. The resting-state fMRI scans were collected using an echo planar imaging sequence with the following parameters: resolution = 2.2 mm isotropic, TE = 35 ms, TR = 2000 ms, flip = 80°, FOV = 207×207 mm, matrix = 94 × 94, 75 slices. In addition, T2-weighted scans were acquired using a 3 T MRI scanner (Discovery MR750, GE Healthcare, Milwaukee, USA) equipped with an 8-channel head coil (GE Healthcare) for the purpose of visualizing and tracing brain lesions. The sequence parameters were as follows: axial scans, slice thickness = 2 mm, slice interval = 2 mm, TE = 98 ms, TR = 6279 ms, flip = 111°, FOV = 240 × 240 mm, matrix = 288 × 3 84, 31 slices; Coronal T2-weighted scan, slice thickness = 2 mm, slice interval = 2 mm, TE = 98 ms, TR = 6264 ms, flip = 111°, FOV = 240 × 240 mm, matrix = 224 × 384, 25 slices; Sagittal T2-weighted scan, slice thickness = 2 mm /slice interval = 2 mm, TE = 98 ms, TR = 6268 ms, flip = 111°, FOV = 240 × 240 mm, matrix = 288 × 384, 31 slices.

In the GSP dataset, one structural scan and two resting-state fMRI scans (6 min and 12 s per scan) were collected for each participant^52^. Data was acquired on matched 3 T Tim Trio scanners (Siemens, Erlangen, Germany) with a 12-channel phased-array head coil. The structural scan consisted in a high-resolution multi-echo T1-weighted magnetization-prepared gradient-echo image with the following parameters: resolution = 1.2 mm isotropic, TI = 1100 ms, TE = 1.54 ms for image 1–7.01 ms for image 4, TR = 2200 ms, flip=7°, FOV = 230, 47 slices. The resting-state fMRI scans were collected using a gradient-echo echo-planar imaging pulse sequence with the following parameters: Resolution=3 mm isotropic, TE = 30 ms, TR = 3000 ms, flip = 85°, FOV = 216, 47 slices with interleaved acquisition and no gap between slices. Participants were asked to keep their eyes open, remain awake, and minimize head movement.

### MRI data processing

#### T1-weighted structural MRI

We extracted the brain from the uniform T1-weighted image produced by the MP2RAGE sequence. To do so, we (i) cropped the neck from one of the gradient echo images (INV2) produced by the MP2RAGE sequence, using FMRIB Software Library (FSL)^53^; (ii) extracted the brain using Advanced Normalization Tools (ANTs)^54^; (iii) generated a brain mask, using ANTs; (iv) dilated the brain mask, using the Connectome Workbench (https://www.humanconnectome.org/software/connectome-workbench); (v) cropped the uniform image produced by the MP2RAGE sequence, using FSL, and vi) applied the brain mask to the uniform image using FSL. Following this, we reconstructed a surface mesh representation of the cortex from each individual participant’s structural image, and registered it to a common spherical coordinate system^55^.

#### T2-weighted structural MRI

We extracted the brain from the T2 scans using FSL and registered the scans to the MNI ICBM152 T2 asymmetric template^56^ using Advanced Normalization Tools (ANTs)^54^.

#### Functional MRI

Preprocessing involved the following steps: (i) deletion of the first four volumes; (ii) slice-timing correction using Statistical Parametric Mapping 2 (SPM)^57^; (iii) motion correction using FSL; (iv) registration of fMRI images to the T1-weighted structural image using FreeSurfer^58^; (v) normalization of global mean signal intensity across runs, using SPM; (vi) bandpass filtering (0.01–0.08 Hz) and regression of head motion, whole-brain global signal, and white matter and ventricular signal, in a single step, using FreeSurfer; (vii) registration to the MNI ICBM 152 T1 asymmetric template; (viii) smoothing using a 6-mm full-width half-maximum kernel, with FreeSurfer; and (ix) projection to surface space, using the FreeSurfer template consisting of 40,962 vertices in each hemisphere.

### Behavioral analysis

The CRST and UPDRS sub-items for the target hand were used as outcome variables for tremor. We determined whether there was an effect of time on these variables using a repeated measures analysis of variance (ANOVA). Post-hoc tests were conducted to compare performance on these tests at baseline and at each of the other time points (1 day, 1 month, 6 months, 12 months), with Sidak correction for multiple comparisons. The same analyses were performed for the non-target hand. Because MRgFUS is a relatively new procedure, we opted for 2-tailed statistical tests with 95% confidence intervals to ensure high confidence in the results. Paired *t*-tests were used to determine whether there was a change in depressive symptoms (BDI) from baseline at 1 month and at long-term follow-up (12 months). SPSS 20 was used to conduct all statistical analyses (IBM, New York).

### MRI analysis

#### Motor thalamus

To identify the motor thalamus, we calculated the FC between the bilateral motor cortex and the thalamus, using the GSP dataset, and found the region in the thalamus with the highest connectivity. The primary motor cortex was segmented using the Destrieux atlas^59^.

#### Functional parcellation of the brain

For each scanning session, we concatenated the five fMRI runs. Using the Desikan-Killiany atlas^60^, we segmented each hemisphere into five zones: the prefrontal, temporal, parietal, occipital, and sensorimotor cortices (consisting of pre-, post- and para-central sulcus regions). Each zone was parcellated separately to capture local fine-grained functional networks using a k-means clustering approach based on FC profiles. FC was estimated by calculating Pearson correlations between the time series of each vertex and all other vertices on the cortical surface. To derive an individual-level parcellation of the cerebral cortex, the cluster boundaries were gradually refined using an iterative approach, described elsewhere^61,62^. The parcellation consisted of 108 clusters in the left hemisphere and 105 clusters in the right hemisphere.

In order to investigate MRgFUS effects on a larger scale, we also parcellated the cortex into the seven canonical large-scale functional networks^35^, which included the sensorimotor network (MOT), default network (DN), limbic network (LMB), visual network (VIS), ventral attention network (VAN), dorsal attention network (DAN), and frontoparietal network (FPN). The parcels identified during the population-level, fine-grained parcellation were assigned to the network with maximal Dice coefficient across the seven canonical functional networks.

#### FC change

We investigated the effect of the MRgFUS intervention on the functional organization of the brain by determining which brain areas exhibited a change in their FC following VIM ablation. We calculated the FC of each cortical parcel with that of the other 212 cortical parcels (FC profile) before and 1 month after the intervention. We calculated FC change as the dissimilarity (1 – Pearson’s correlation *r*) between each parcel’s pre- and post-FC profile for each participant. To ensure that the FC change variable was not simply a reflection of within-individual variability in FC, we regressed out within-individual variability using general linear regression. Within-individual variability for each parcel was determined by calculating its FC dissimilarity between the first and second half of each concatenated scanning session, and averaging the dissimilarity across the pre- and post-sessions to give us an average value of within-subject variability for each parcel and participant. The residual value (plus the constant) was taken as the final estimate of FC change. We also investigated FC change at the level of the large-scale functional networks. For each network and parcel, we performed one-sample *t*-tests to verify whether FC change was larger than 0. For these analyses, we did not expect statistically significant findings due to the small patient sample size (*N* = 13) and because the lesions were small (Table 1). Therefore, brain regions were deemed to have experienced a change in FC if their amount of change, calculated using the regression described above, exceeded the mean of all regions. *P* values are nonetheless reported.

We also sought to determine whether the regions that were most affected by the intervention were also the regions that are functionally connected to the lesion target. To calculate lesion site connectivity, we used the control group to obtain normative FC based on each of the 13 patient seeds. The final connectivity map was averaged across seeds. We binarized the individual functional parcels according to whether they were functionally connected to the lesion site (*r* > 0) or not (*r* ≤ 0) in the normative map, and according to whether they demonstrated a change in FC above the mean in patients. We quantified the overlap between these two sets of regions (*x* and *y*) using the Dice overlap coefficient:

1 $${Dice}=\frac{2{\rm{|}}x\cap y{\rm{|}}}{\left|x\right|+{\rm{|}}y{\rm{|}}}$$

Of the overlap regions, we assessed the proportion of regions that belonged to each large-scale functional network.

Finally, we investigated the association between tremor alleviation and FC change within the brain’s cortical hand region. We performed Spearman correlations covaried with baseline tremor, age, and sex.

#### Similarity with normative FC

We investigated the pattern of FC of patients’ cortical target hand region, and whether it was more similar to that of the control group (normative FC) following the MRgFUS intervention. We calculated the similarity between the parcel’s FC profile in each patient and the average FC profile of the same parcel in the healthy control group’s left hemisphere using Pearson correlations. We compared pre- and post-intervention similarities using a paired *t*-test, with one-sided 95% confidence intervals for the cortical hand region as these analyses were hypothesis-driven, and two-sided intervals for the control regions, which included regions randomly selected in the ventral attention network, dorsal attention network, default network, and frontoparietal network.

### Reporting summary

Further information on research design is available in the Nature Research Reporting Summary linked to this article.

## Supplementary information

Reporting Summary

## Data Availability

The GSP dataset is available at: http://neuroinformatics.harvard.edu/gsp/. The patient and control data that support the findings of this study are available from the corresponding authors upon request.

## Code availability

The code used in this article is available at http://nmr.mgh.harvard.edu/bid/DownLoad.html.
