# Supplementary material for: Focused ultrasound thalamotomy for tremor treatment impacts the cerebello-thalamo-cortical network
Source: NPJ Parkinsons Dis. 2023 Jun 15;9:90. doi: 10.1038/s41531-023-00543-8 (PMC10272231; doi:10.1038/s41531-023-00543-8)
Supplement: Supplementary file 1 — Reporting Summary [file 41531_2023_543_MOESM1_ESM.pdf]

nature portfolio

Corresponding author(s): Hesheng Liu, Meiyun Wang

Last updated by author(s): 2023/04/21

Reporting Summary

Nature Portfolio wishes to improve the reproducibility of the work that we publish. This form provides structure for consistency and transparency in reporting. For further information on Nature Portfolio policies, see our Editorial Policies and the Editorial Policy Checklist.

Statistics

For all statistical analyses, confirm that the following items are present in the figure legend, table legend, main text, or Methods section.

n/a	Confirmed	
☐	☒	The exact sample size (n) for each experimental group/condition, given as a discrete number and unit of measurement
☐	☒	A statement on whether measurements were taken from distinct samples or whether the same sample was measured repeatedly
☐	☒	The statistical test(s) used AND whether they are one- or two-sided<br>*Only common tests should be described solely by name; describe more complex techniques in the Methods section.*
☐	☒	A description of all covariates tested
☐	☒	A description of any assumptions or corrections, such as tests of normality and adjustment for multiple comparisons
☐	☒	A full description of the statistical parameters including central tendency (e.g. means) or other basic estimates (e.g. regression coefficient) AND variation (e.g. standard deviation) or associated estimates of uncertainty (e.g. confidence intervals)
☐	☒	For null hypothesis testing, the test statistic (e.g. F, t, r) with confidence intervals, effect sizes, degrees of freedom and P value noted<br>*Give P values as exact values whenever suitable.*
☒	☐	For Bayesian analysis, information on the choice of priors and Markov chain Monte Carlo settings
☒	☐	For hierarchical and complex designs, identification of the appropriate level for tests and full reporting of outcomes
☐	☒	Estimates of effect sizes (e.g. Cohen's d, Pearson's r), indicating how they were calculated

Our web collection on statistics for biologists contains articles on many of the points above.

Software and code

Policy information about availability of computer code

Data collection	No code was used to collect the data.
Data analysis	The data was analyzed using Matlab R2018b (Mathworks) and SPSS Statistics 20.0.

For manuscripts utilizing custom algorithms or software that are central to the research but not yet described in published literature, software must be made available to editors and reviewers. We strongly encourage code deposition in a community repository (e.g. GitHub). See the Nature Portfolio guidelines for submitting code & software for further information.

Data

Policy information about availability of data

All manuscripts must include a data availability statement. This statement should provide the following information, where applicable:

- Accession codes, unique identifiers, or web links for publicly available datasets
- A description of any restrictions on data availability
- For clinical datasets or third party data, please ensure that the statement adheres to our policy

The data that support the findings of this study are available from the corresponding authors upon reasonable request.

Research involving human participants, their data, or biological material

Policy information about studies with human participants or human data. See also policy information about sex, gender (identity/presentation), and sexual orientation and race, ethnicity and racism.

Reporting on sex and gender	Sex was not considered in the study design, nor was it in the analyses, except to ensure that groups were matched based on sex. This was because patients were enrolled on a rolling, consecutive basis until up to 10 patients in each diagnosis group were recruited, regardless of sex. We did not expect sex to play a role in the functional reorganization of the brain following lesioning of the ventral intermediate nucleus of the thalamus, therefore sex was not investigated as a modulating factor. Sex was self-reported. Disaggregated sex and gender data were not collected.
Reporting on race, ethnicity, or other socially relevant groupings	There were no socially constructed or socially relevant categorization variables in this study.
Population characteristics	The patient and healthy control group were matched on age, sex, and cognitive status.<br>The patients in the patient group had either a tremor-dominant Parkinson's disease diagnosis, or an Essential Tremor diagnosis. The healthy control group had no history of neurologic or psychiatric disorders.
Recruitment	10 patients with tremor-dominant Parkinson's disease and three patients with Essential Tremor were referred to the study due to uncontrolled and medication-resistant tremor. Functional reorganization of the brain following brain lesioning are unlikely to be affected by self-bias.
Ethics oversight	Henan Provincial People's Hospital Institutional Review Board.

Note that full information on the approval of the study protocol must also be provided in the manuscript.

Field-specific reporting

Please select the one below that is the best fit for your research. If you are not sure, read the appropriate sections before making your selection.

☒ Life sciences ☐ Behavioural & social sciences ☐ Ecological, evolutionary & environmental sciences

For a reference copy of the document with all sections, see nature.com/documents/nr-reporting-summary-flat.pdf

Life sciences study design

All studies must disclose on these points even when the disclosure is negative.

Sample size	For this clinical trial, a statistical sample size analysis was not proposed; instead, 100 patients with medication-refractory TDPD (https://clinicaltrials.gov/ct2/show/NCT04002596) and 100 patients with ET (https://clinicaltrials.gov/ct2/show/NCT03253991) were planned to be enrolled. The current study represents one of the first in China to administer MRgFUS treatment. Therefore, before enrolling large numbers of participants, we first had to assess the success of the therapy on a small sample of patients.
Data exclusions	There were no data exclusions.
Replication	The current study investigates a novel treatment for tremor in China. Due to this, the safety and clinical success of the treatment first had to be established, therefore few patients were enrolled, precluding the opportunity for replication. Replication of our findings will be investigated once more patients undergo VIM-MRgFUS treatment.
Randomization	Allocation into groups (patients vs controls) was not random. The current study sought to determine the effects of a novel treatment for tremor in China, therefore randomized allocation is not appropriate.
Blinding	Blinding was not relevant to the study. Neuroimaging methods are automated and therefore not prone to bias. The effects of the treatment were determined in patients by comparing follow-up scores to baseline scores, therefore group allocation-based biases were not in play.

Reporting for specific materials, systems and methods

We require information from authors about some types of materials, experimental systems and methods used in many studies. Here, indicate whether each material, system or method listed is relevant to your study. If you are not sure if a list item applies to your research, read the appropriate section before selecting a response.

Materials & experimental systems	
n/a	Involved in the study
☒	☐ Antibodies
☒	☐ Eukaryotic cell lines
☒	☐ Palaeontology and archaeology
☒	☐ Animals and other organisms
☐	☒ Clinical data
☒	☐ Dual use research of concern
☒	☐ Plants

Methods	
n/a	Involved in the study
☒	☐ ChIP-seq
☒	☐ Flow cytometry
☐	☒ MRI-based neuroimaging

Clinical data

Policy information about clinical studies

All manuscripts should comply with the ICMJE guidelines for publication of clinical research and a completed CONSORT checklist must be included with all submissions.

Clinical trial registration	https://clinicaltrials.gov/ct2/show/NCT04002596; https://clinicaltrials.gov/ct2/show/NCT03253991
Study protocol	See links above.
Data collection	Patients recruited between February and April 2019; initial testing took place between February and September 2019, and follow-up visits occurred until September 2020. Recruitment and testing took place in a hospital setting.
Outcomes	All patients were administered the Fahn-Tolosa-Marin Clinical Rating Scale for Tremor (CRST). Because patients with TDPD mostly experience tremor at rest, and patients with ET mostly during actions, we selected the sum of the "Tremor at rest, with posture holding, and with action and intention" of the target upper extremity scores in Part A as the outcome measure. Patients with TDPD, but not ET, were administered the Unified Parkinson's Disease Rating Scale (UPDRS). Because these patients mostly experience tremor at rest, we selected the "Rest tremor amplitude" in the target upper limb in Part III (motor examination) as the outcome measure. The Beck Depression Inventory (BDI) was administered to assess depression at each time point. However, the patients with ET were not administered the BDI at the 6-month and 12-month follow-ups.

Magnetic resonance imaging

Experimental design

Design type	Resting-state
Design specifications	For patients, at the baseline and one-month follow-up visits, one structural scan (8min 50sec) and 5 resting-state scans (6min 14sec each for a total duration of 31min 10sec) were performed. Healthy control participants performed a single visit; the same MRI design was used.<br>In the GSP dataset, one structural scan and two resting-state fMRI scans (6 min and 12 s per scan) were collected for each participant.
Behavioral performance measures	No variables were recorded during scanning as participants were only asked to rest while lying in the scanner.

Acquisition

Imaging type(s)	Functional, structural.
Field strength	3T
Sequence & imaging parameters	MRI data were collected using a 3T MRI scanner (Magnetom Prisma, Siemens Healthcare, Erlangen, Germany) equipped with a 64-channel head coil. T1-weighted structural scans were acquired using a gradient echo MP2RAGE sequence with the following parameters: resolution=1 mm isotropic, TI1=755 ms, TI2=2500 ms, TE=3.43 ms, TR=5000 ms, flip1=4°, flip2=5°, bandwidth=240 Hz/pix, echo spacing=7.1 ms, matrix=256×256, 208 slices, acceleration factor of 3 (32 reference lines) in the primary phase encoding direction and online GRAPPA image reconstruction. The resting-state fMRI scans were collected using an echo planar imaging sequence with the following parameters: resolution= 2.2 mm isotropic, TE=35 ms, TR=2000 ms, flip=80º, FOV=207x207 mm, matrix=94x94, 75 slices. In addition, T2-weighted scans were acquired using a 3T MRI scanner (Discovery MR750, GE Healthcare, Milwaukee, USA) equipped with an 8-channel head coil (GE Healthcare) for the purpose of visualizing and tracing brain lesions. The sequence parameters were as follows: axial scans, slice thickness=2 mm, slice interval=2 mm, TE= 98 ms, TR=6279 ms, flip=111°, FOV=240x240 mm, matrix=288×384, 31 slices; Coronal T2-weighted scan, slice thickness=2 mm, slice interval=2 mm, TE=98 ms, TR=6264 ms, flip=111°, FOV=240x240 mm, matrix=224×384, 25 slices; Sagittal T2-weighted scan, slice thickness=2 mm /slice interval=2 mm, TE=98 ms, TR=6268 ms, flip=111°, FOV=240x240 mm, matrix=288×384, 31 slices.<br>In the GSP dataset, one structural scan and two resting-state fMRI scans (6 min and 12 s per scan) were collected for each participant52. Data was acquired on matched 3T Tim Trio scanners (Siemens, Erlangen, Germany) with a 12-channel phased-array head coil. The structural scan consisted in a high-resolution multi-echo T1-weighted magnetization-prepared gradient-echo image with the following parameters: resolution=1.2 mm isotropic, TI=1100 ms,

TE=1.54 ms for image 1 to 7.01 ms for image 4, TR=2200 ms, flip=7°, FOV=230, 47 slices. The resting-state fMRI scans were collected using a gradient-echo echo-planar imaging pulse sequence with the following parameters: resolution=3 mm isotropic, TE=30 ms, TR=3000 ms, flip=85°, FOV=216, 47 slices with interleaved acquisition and no gap between slices.

Area of acquisition: Whole-brain scans.

Diffusion MRI: ☐ Used ☒ Not used

Preprocessing

Preprocessing software: T1-weighted structural MRI. We extracted the brain from the uniform T1-weighted image produced by the MP2RAGE sequence. To do so, we i) cropped the neck from one of the gradient echo images (INV2) produced by the MP2RAGE sequence, using FMRIB Software Library (FSL); ii) extracted the brain using Advanced Normalization Tools (ANTs); iii) generated a brain mask, using ANTs; iv) dilated the brain mask, using the Connectome Workbench (https://www.humanconnectome.org/software/connectome-workbench); v) cropped the uniform image produced by the MP2RAGE sequence, using FSL, and vi) applied the brain mask to the uniform image using FSL. Following this, we reconstructed a surface mesh representation of the cortex from each individual participant's structural image, and registered it to a common spherical coordinate system.
T2-weighted structural MRI. We extracted the brain from the T2 scans using FSL and registered the scans to the MNI ICBM152 T2 asymmetric template using Advanced Normalization Tools (ANTs).
Functional MRI. Preprocessing involved the following steps: i) deletion of the first four volumes; ii) slice-timing correction using Statistical Parametric Mapping 2 (SPM); iii) motion correction using FSL; iv) registration of fMRI images to the T1-weighted structural image using FreeSurfer; v) normalization of global mean signal intensity across runs, using SPM; vi) bandpass filtering (0.01-0.08 Hz) using FreeSurfer; vii) bandpass filtering (0.01-0.08 Hz) and regression of head motion, whole-brain global signal, and white matter and ventricular signal, in a single step, using FreeSurfer; viii) registration to the MNI ICBM 152 T1 asymmetric template; ix) smoothing using a 6-mm full-width half-maximum kernel, with FreeSurfer; and x) projection to surface space, using the FreeSurfer template consisting of 40,962 vertices in each hemisphere.

Normalization: Non-linear normalization.

Normalization template: We registered each individual's fMRI data to the FreeSurfer template which consisted of 40,962 vertices in each hemisphere.

Noise and artifact removal: We regressed out head-motion , whole-brain signal , and ventricular and white-matter signal.

Volume censoring: The first four volumes of each fMRI scan were deleted.

Statistical modeling & inference

Model type and settings: For each network and parcel, we performed one-sample t-tests to verify whether FC change was larger than 0. We compared pre- and post-intervention similarities using a paired t-test, with one-sided 95% confidence intervals for the cortical hand region as these analyses were hypothesis-driven, and two-sided intervals for the control regions.

Effect(s) tested: One-sample and paired samples t-tests were used (see above).

Specify type of analysis: ☐ Whole brain ☐ ROI-based ☒ Both

Anatomical location(s): Using the Desikan-Killiany atlas, we segmented each hemisphere into five zones: the prefrontal, temporal, parietal, occipital, and sensorimotor cortices (consisting of pre-, post- and para-central sulcus regions). Each zone was parcellated separately to capture local fine-grained functional networks using a k-means clustering approach based on FC profiles. FC was estimated by calculating Pearson correlations between the time series of each vertex and all other vertices on the cortical surface. To derive an individual-level parcellation of the cerebral cortex, the cluster boundaries were gradually refined using an iterative approach, described elsewhere. The parcellation consisted of 108 clusters in the left hemisphere and 105 clusters in the right hemisphere.

Statistic type for inference (See Eklund et al. 2016): *Specify voxel-wise or cluster-wise and report all relevant parameters for cluster-wise methods.*

Correction: FDR q=0.05

Models & analysis

n/a	Involved in the study
☐	☒ Functional and/or effective connectivity
☒	☐ Graph analysis
☒	☐ Multivariate modeling or predictive analysis

Functional and/or effective connectivity: Pearson correlations.
